# Triple Negative Breast Cancer – An Overview

**DOI:** 10.4172/2161-1041.S2-001

**Published:** 2013

**Authors:** Kartik Aysola, Akshata Desai, Crystal Welch, Jingyao Xu, Yunlong Qin, Vaishali Reddy, Roland Matthews, Charlotte Owens, Joel Okoli, Derrick J Beech, Chandrika J Piyathilake, Shyam P Reddy, Veena N Rao

**Affiliations:** 1 Cancer Biology Program, Department of OB/GYN, Morehouse School of Medicine, Georgia Cancer Center for Excellence, Grady Health System, Atlanta, GA 30303, USA; 2 Department of Surgery, Morehouse School of Medicine, Grady Health System, Atlanta, GA 30303, USA; 3 Department of Internal Medicine, University of Buffalo, Erie County Medical Center, 462 Grider St, Buffalo NY 14215, USA; 4 Wallace Tumour Institute 420 D, 1824 6th Avenue South, University of Alabama at Birmingham, Birmingham AL 35294, USA

**Keywords:** TNBC, BRCA1, CISPLATIN, EGFR, PARP, Hedgehog, NOTCH, BCL-2, UBC9, WNT/B-CATENIN

## Abstract

Triple Negative Breast Cancer (TNBC) is a heterogeneous disease that based on immunohistochemistry (IHC) is estrogen receptor (ER) negative, progesterone receptor (PR) negative and human epidermal growth factor receptor 2 (HER2) negative. TNBC is typically observed in young AA women and Hispanic women who carry a mutation in the BRCA1 gene. TNBC is characterized by a distinct molecular profile, aggressive nature and lack of targeted therapies. The purpose of this article is to review the current and future novel signalling pathways as therapeutic approaches to TNBC. Recent Identification of a new BRCA1 trafficking pathway holds promise in the future for the development of targeted therapies for TNBC.

Triple Negative Breast Cancer (TNBC) is a subtype of breast cancer that based on immunohistochemistry (IHC) is estrogens receptor (ER) negative, progesterone receptor (PR) negative and human epidermal growth factor receptor 2 (HER2) negative [1]. TNBC is characterized by its unique molecular profile, aggressive nature, distinct metastatic patterns and lack of targeted therapies. It is estimated that out of the worldwide breast cancer burden, approximately 170,000 cases are TNBC and account for ~10-20% of invasive breast cancers [1,2].

## Molecular Profile and IHC Phenotype

Breast cancers are typically classified into seven subtypes (3): luminal A (ER positive and histologic low grade), luminal B (ER positive and histologic high grade), HER2 overexpressing, basal-like (2 types – BL1 and BL2), immunomodulatory (IM), mesenchymal (M), mesenchymal stem-like (MSL) [3] and normal breast-like tumours [1]. Majority of the TNBC are the basal-like subtype, and many basal-like breast cancers are triple negative; they are not equivalent in terms of gene expression signatures and IHC analysis [4]. Basal-like breast cancer is a classification based on gene expression profiling. Although they appear to be synonymous, there is up to 30% discordance between the two groups [5,6]. In addition to low expression of ER, PR and HER2, basal-like breast cancers are characterized by a high expression of CK5, CK14, caveolin-1, caix, p63, EGFR (Epidermal Growth Factor Receptor)/HER1, which reflects on the mammary gland basal/myoepithelial cell component [7].

Additionally, several proteins integrally involved in DNA repair are aberrantly expressed in TNBC, which may have implications on sensitivity to chemotherapeutic agents like, platinum-based drugs. High p53 IHC expression is common in basal-like breast cancer [8]. Several additional and targetable molecular pathways implicated in the pathogenesis of basal-like breast cancer include the mutagen activated protein (MAP) kinase pathway, the Akt pathway, and the poly ADP-ribose polymerase1 (PARP1) pathway [9].

## Association with BRCA1 Mutation status

It has been observed that a high percentage of BRCA1- associated hereditary and sporadic breast cancers are triple negative and express a high proportion of basal like cytokeratins (CK5,14,17), as well as P-Cadherin and HER1/EGFR [10,11]. Gene expression studies support this association among patients with BRCA1 mutations that breast tumours tend to cluster within the basal like category [12].

## Epidemiology and Risk Factors

Several large scale population based studies indicate that TNBC are up to three times more likely to occur among pre-menopausal women of African-American descent [13]. Certain epidemiologic studies like the Carolina Breast Cancer Study illustrated that as compared to the luminal a tumours, basal-like tumours were more likely to arise among women with early menarche, higher parity, younger age at full term pregnancy, and shorter duration of breast feeding, higher body mass index, and higher waist to hip ratio, especially among pre-menopausal patients [14]. Another study done by Bauer et al. [13] found that younger, non-Hispanic black and Hispanic women diagnosed with TNBC, had tumours that were more aggressive, and these women had poorer survival regardless of stage. In addition, non-Hispanic black women with late stage TNBC had the poorest survival of any comparable group [13].

## Clinical Characteristics

TNBC is well known for its aggressive behaviour and is characterized by onset at a younger age, high mean tumour size, higher grade tumours and sometimes, a higher rate of node positivity [15]. Additionally, this group is also known for an early peak of recurrence between the first and third year after diagnosis, and more aggressive metastases which are more likely to occur in viscera particularly in the lungs and brain, and less likely to spread to the bone [16]. Based upon histological findings the majority of the triple negative breast cancers are of ductal origin; however, several other aggressive phenotypes also appear to be over represented, including metaplastic, apocrine and adenoid cystic [17]. A histological study of basal-like tumours, all being ER/HER2 negative, yielded marked increase in mitotic count, geographic necrosis, pushing borders of invasion and stromal lymphocytic response [18].

## Prognosis

An inferior prognosis in the basal-like breast cancer, as compared to the luminal type, has been uniformly demonstrated by a variety of studies [19]. Population based studies have also demonstrated similar results with reduced breast cancer specific survival among those with TNBC, as compared with the luminal subtype [14].

A recently reported Canadian series [15] evaluating prognosis in over 1,500 women, showed an increased likelihood of distant recurrence and death among women with triple negative breast cancer, as compared to the non triple negative disease. Studies have consistently shown that more aggressive visceral and soft tissue relapses are more common and bone relapses less common among those diagnosed with triple negative versus ER positive disease [20]. An estimated 15% of all patients with breast cancer will develop brain metastasis. In a series of over 3,000 patients with brain metastasis arising from breast cancer treated from 1989 to 2006, multivariate analysis indicated that triple negative status was the greatest risk factor for the development of cerebral metastasis (odds ratio=4.16; p<0.001), above that of HER2 positive status (OR=3.43; p=0.005) [21]. In another study using patients with BRCA1 mutation, there was 82% complete pathologic response in those treated with Cisplatinum alone [22].

## Therapeutic Strategies

Although triple negative breast cancers are associated with a generally poor breast cancer specific outcome, most are not resistant to chemotherapy. These patients have an extremely poor prognosis and relapse and die quickly. Several therapies are being developed that target specific biomarkers of TNBC or basal-like subtype [23]. These strategies include EGFR-targeted agents, androgen receptor targeted agents, anti-antigenic agents, and PARP inhibitors are offering an option in triple negative disease; however, their uses as of date are limited to clinical trials and more work is needed to identify targets that yield high therapeutic ratios [23]. TNBC with BRCA1 gene mutations may be more sensitive to agents that cause DNA damage, such as Cisplatin [24]. Other, more recent promising therapeutic targets for TNBC include the NOTCH, Hedgehog and Wnt/b-Catenin signalling pathways [2]. Studies have shown that these therapies alter the apoptotic pathway, inhibiting tumour progression [2].

## Chemotherapy

Adjuvant chemotherapy has been shown to not only prolong disease-free survival in patients but overall survival as a whole; however, TNBC lacks the typical targeted receptors found in luminal or HER-2 disease and therefore cannot be treated with hormonal agents, such as SERMS, aromatise inhibitors or HER2 antagonists [25]. To combat this issue several neoadjuvant studies have been done which accentuated the relationship between chemo sensitivity and outcome, revealing proportionally higher sensitivity to anthracycline or anthracycline/taxane–based chemotherapy such as Doxorubicin and Cyclophosphamide (standard chemotherapeutic agents), for basal like/ER negative breast cancers as compared to the luminal subtype [26]. Highest response rates were noticed among those classified as basal like (85%) and HER2 positive (70%), as compared to luminal (47%, p<0.0001). Despite initial chemo sensitivity, disease free survival (p=0.04) and overall survival (p=0.02) remained poorest among those with basal like and HER2 positive tumours, compared to the luminal subtype [26]. Although triple negative disease is highly responsive to Anthracycline/Taxane chemotherapy treatment, a high risk of relapse still remains if the tumour is not completely eradicated.

Additionally, pre-clinical and clinical studies indicate that tumours with BRCA1 dysfunction are sensitive to platinum agents such as Cisplatin, and Carboplatin which function by causing DNA damage and promote tumour cell apoptosis [25,27]. Studies have shown that p63, a family member of p53, is responsible for controlling a survival pathway that directly mediates Cisplatin sensitivity in TNBC; it therefore, can possibly be used as a biomarker to predict response to platinum therapy in triple negative breast cancer. However, this finding warrants further investigation and currently platinum’s are not recommended for the adjuvant treatment of TNBC [25].

## EGFR Inhibitors

EGFR expression is approximately seen in 60% of triple negative breast tumors, thus providing a reasonable targeted treatment approach [6]. A phase II trial evaluating the combination of Cetuximab (a chimeric monoclonal antibody targeting EGFR) and Carboplatin, weekly for 3 to 4 weeks reported a response rate of 18% and overall clinical benefit of 27% among 102 patients with advanced TNBC [28].

Another study evaluating the combination of Irinotecan and Carboplatin with or without Cetuximab reported response rates of 49% and 30% respectively, among 72 patients with pre-treated TNBC. EGFR inhibitor Panitumumab, when used in combination with standard chemotherapeutic agents for neoadjuvant therapy for inoperable TNBC was recently reported to have shown a pathological complete response rate of 65% [25]. Other studies have recently demonstrated that EGFR inhibitors when used in combination with Texans or platinum’s may increase the efficacy of the other agents [25]. To date, the majority of data with EGFR inhibitors has generally been interpreted as negative.

## PARP Inhibitors

PARP1, a gene that encodes an enzyme involved in the molecular events leading to cell recovery from DNA damage, when inhibited, leads to the accumulation of double-stranded DNA breaks. Cells deficient in BRCA1 and BRCA2 (required for normal homologous recombination), are exquisitely sensitive to PARP1 inhibition. Several PARP inhibitors Olaparib, Velaparib and PF-01367338 are currently in clinical trials and hold a promising future [25]. The study demonstrated a statistically significant 50% reduction in the risk of death; however, phase III trials failed to show statistically significant benefit for this combination, hence, the drug has been discontinued but some biomarker analysis is still underway to determine if a specific subset of patient may benefit from the drug [16,28,29]. This therefore highlights the need for continued research and clinical trials.

## Antiangiogenic Agents

The antiangiogenic agent Bevacizumab, a monoclonal antibody that targets all forms of VEGF, has been evaluated in a number of large phase III trials as treatment of metastatic breast cancer [25]. The landmark study E2100 illustrated improvement in progressionfree survival (11.8 vs. 5.9 months, HR=0.60, *p*<0.001) when adding Bevacizumab to Paclitaxel chemotherapy compared with single-agent Paclitaxel alone in first-line treatment of metastatic disease [25]. This subsequently led to subgroup analysis that demonstrated similar progression free survival benefits in both patients with TNBC and non-TNBC with the use of Bevacizumab plus a taxane [25]. Currently, the BEATRICE trial is prospectively investigating this combination as adjuvant therapy in TNBC [25]. Additionally, several small-molecule inhibitors of the VEGF pathway appear to have activity in the subset of pre-treated triple-negative breast cancer and definitive studies showing overall survival benefit will be needed prior to re approval [30,31].

## Conclusion

In conclusion, TNBC is a difficult and complex disease entity that is both confusing and frustrating for researchers, physicians and patients. To date there are multiple approaches attempting to improve care of triple negative breast cancer patients, including DNA damaging agents like platinum’s, targeted EGFR and VEGF inhibitors, and, PARP inhibitors; however, none have been as clinically successful as anticipated and more targeted therapies need to be developed and explored. The Wnt/b-Catenin, NOTCH and Hedgehog signaling pathways are being considered as novel therapeutic targets for TNBC [2].

## Future Direction

The key to developing targeted therapies for TNBC will depend on understanding the molecular mechanism for the development of these cancers. There are seminal studies by Hosey et al. [32] and by our group [33,34] (unpublished results) on the possible molecular mechanisms of BRCA1 dysfunction which could result in ER negative breast cancers. Recently Ubc9, a sole SUMO-E2-conjugating enzyme for sumoylation was found to bind BRCA1 proteins and ferry it to the nucleus unlike disease associated mutant BRCA1 proteins [34]. Over expression of Ubc9 was observed in breast tumours and TNBC cell lines [34]. BRCA1 function-based cellular assays have been designed to detect loss of binding to Ubc9 by BRCA1 mutants (Patent number US 8,372,580). These assays not only predict the risk for developing TNBC but may also lead to the development of targeted therapies for hereditary and sporadic TNBC.
